# Prevalence of Parasomnia in Autistic Children with Sleep Disorders

**DOI:** 10.4137/cmped.s1139

**Published:** 2009-01-22

**Authors:** Xue Ming, Ye-Ming Sun, Roberto V. Nachajon, Michael Brimacombe, Arthur S. Walters

**Affiliations:** 1 Department of Neuroscience, UMDNJ-New Jersey Medical School, Newark, NJ.; 2 Creedmoor Psychiatric Center, Queens Village, NY and Department of Psychiatry, Columbia University Medical Center, New York, NY.; 3 Pediatric Sleep Disorders Center, St. Joseph Children’s Hospital, Paterson, NJ.; 4 Sleep/Wake Center, Palisades General Medical Center, North Bergen, NJ.; 5 Department of Preventive Medicine, UMDNJ-New Jersey Medical School, Newark, NJ.; 6 Center for Sleep Disorders Treatment, Research and Education, New Jersey Neuroscience Institute at JFK Medical Center, Seton Hall University, School of Graduate Medical Education, Edison, NJ.

**Keywords:** Autism spectrum disorders, parasomnia, sleep terror, confusional arousal, disorders of partial arousal

## Abstract

The prevalence of sleep related complaints is reported by questionnaire studies to be as high as 83.3% in children with autism spectrum disorders (ASD). Questionnaire studies report the presence of various parasomnia in ASD. However, no polysomnographic study reports non-REM parasomnias and only a single study reports REM related parasomnias in ASD. We investigated the prevalence and characteristics of sleep disorders by polysomnographic study and questionnaires in a cohort of 23 children with ASD and 23 age-matched children of a non-autistic comparison group. The results showed significantly more non-REM parasomnias in 14 children with ASD on polysomnograms (PSG) and 16 ASD children by questionnaire, a finding that was not associated with medication use, other comorbid medical or psychiatric disorders, or sleep disordered breathing. Of the 14 children with ASD who had PSG evidence of parasomnia, 11 of them had a history suggestive of parasomnia by questionnaire. There was a high sensitivity but a low specificity of parasomnia in ASD by questionnaire in predicting the presence of parasomnia in the PSG. Of the parasomnias recorded in the laboratory, 13 ASD children had Disorders of Partial Arousal, consistent with sleep terrors or confusional arousals. Furthermore, multiple episodes of partial arousal occurred in 11 of the 13 ASD children who had PSG evidence of Disorders of Partial Arousal. Of the 11 ASD children with multiple episodes of partial arousal, 6 ASD children had multiple partial arousals during both nights’ PSG study. Sleep architecture was abnormal in children with ASD, characterized by increased spontaneous arousals, prolonged REM latency and reduced REM percentage. These results suggest a high prevalence of parasomnia in this cohort of children with ASD and a careful history intake of symptoms compatible with parasomnia could be prudent to diagnose parasomnia in ASD children when performing a PSG is not possible.

## Introduction

Autism spectrum disorders (ASD) are neurodevelopmental disorders, consisting of autistic disorder, pervasive developmental disorder-not otherwise specified (PDD-NOS) and Asperger’s disorder. Individuals with ASD experience a number of comorbid medical disorders, among which are the presence of sleep disorders.

Children with ASD are frequently reported by history to display problematic sleep patterns which are often severe in nature. The sleep problems include difficulty in settling to sleep, lengthy episodes of night waking with or without confusion, crying or screaming during sleep, bruxism, bedwetting, early morning awakening, shortened night sleep and irregularities of the sleep/wake rhythm.^1^,^2^,^3^ Various sleep disorders including parasomnias were reported in ASD by questionnaire study and the prevalence of parasomnias was as high as more than one fourth of population for a specific parasomnia.^4^ A survey^5^ showed significantly more prevalent and severe sleep problems in children with PDD-NOS with normal intelligence, compared with controls. In addition, the scales for sleep onset delay, sleep duration, sleep anxiety, and parasomnias were significantly higher in the PDD-NOS group in this survey. There are a few PSG studies in ASD individuals,^6^,^7^,^8^,^9^,^10^ most focused on sleep architectures and/or insomnia and these studies did not report the presence or absence of parasomnia with the exception of one study that reported PSG evidence of REM sleep behavior disorder (RBD) in ASD children with sleep complaints.^11^ Although studies investigating sleep disorders in individuals with ASD are increasing, there are only a limited number of studies utilizing both PSG and questionnaires.^8^,^9^,^10^ The aim of this study is to determine systematically the prevalence of specific sleep disorders in ASD children with sleep complaints using both questionnaire and PSG, as well as the value of a questionnaire/clinical history in diagnosing parasomnia when performing PSG is not possible. We report a significant increased prevalence of parasomnias in children with ASD and clinical history pertaining to parasomnia is a reasonable alternative in diagnosing parasomnia.

## Methods

### Participants

Twenty three children with ASD (autistic disorder, PDD-NOS, Asperger’s disorder) with complaints of sleep related problems were enrolled in this study. All ASD children’s clinical manifestation was carefully examined using DSM IV (American Psychiatric Association 1994)^12^ criteria for the diagnosis of ASD. The diagnosis of ASD in 5 children was questionable by DSM IV criteria but was confirmed by the Autism Diagnostic Interview-Revised^13^ and Autism Diagnostic Observation Schedule-Generic.^14^ All the subjects with ASD were evaluated for sleep disorders during 1999–2001 at The Autism Center, UMDNJ. There were a total of 34 ASD children referred for diagnostic PSG studies. The chief complaints of sleep disorders in these children were; difficulty in sleep onset and maintenance (18 children), frequent night time awakening (15 children), and night awakenings with crying or screaming (3 children), noisy sleep (1 child), and restless sleep (2 children). Eight of the families refused PSG study, and three children could not tolerate PSG with resultant termination. These 11 children were excluded from this study. Of the 23 ASD children who participated in both questionnaire and PSG study, there were 10 children with a diagnosis of autistic disorder, 11 children with PDD-NOS, and 2 children with Asperger’s disorder. The neurological and general physical examinations were non-focal in all children with ASD except for the presence of mild hypotonia and repetitive motor mannerisms that are frequently seen in children with ASD. Seventeen of the 23 children with ASD were able to talk in sentences or short sentences, another 5 children with ASD used single words or phrases to communicate, and the remaining one child was nonverbal but could indicate needs by using someone else’s hand to point.

There are few available studies of the prevalence of various sleep disorders and PSG findings in general in children. To compare our study of ASD children with another population, we studied 23 non-autistic children with no known developmental or chronic illness with the exception of past history of acute reactive airway disease and/or allergy in 7 children, who were referred to our sleep laboratory for evaluation of sleep disordered breathing and snoring but who were found not to have sleep disordered breathing on PSG (apnea hypopnea index (AHI) < 1/hour of sleep). These children were selected prospectively and sequentially to serve as a non-autistic comparison group using chart review. The inclusion criteria were for the comparison children between 3–15 years old whose AHI was less than one per hour of sleep on PSG and no history of neurological disorders and no acute illness including reactive airway disease or allergy at time of PSG study. Non-autistic children with an AHI > 1/hour were excluded to eliminate any confounding effects of sleep disordered breathing on sleep architecture. ASD children were not excluded if sleep disordered breathing was present. The characteristics of each group of children including medication intake is listed in Table 1.

### Procedures

Sleep questionnaires including questions on current and past history of insomnia, sleep disordered breathing, parasomnias, sleep hygiene, sleep schedules and bedtime were completed by the parents/guardians of both the non-autistic and ASD children at the night of PSG study. There were a total of 49 questions in the form of Yes/No or checklist answers. There were questions pertaining to the frequency of occurrence, such as “daily, weekly, monthly or yearly”, “never, occasionally, frequently or always.” Some questions required the parents to estimate the average period of a specific parameter, such as sleep onset time, or number of awakening per night. Bedwetting was defined as urinary incontinence during sleep in these children who had persistently acquired bladder control and were not wearing diapers at night. Parental reports of sudden night arousal with confusion or screaming with subsequent resumption of sleep were classified as possible Disorders of Partial Arousal without further specification.

All children with ASD were evaluated polysomnographically for 2 consecutive nights, allowing children with ASD to adjust to the change in sleep environment. Sleep architecture, EEG with full seizure montage (international 10–20 system), respiration, periodic limb movement during sleep (PLMS) and parasomnia activity were evaluated by standard PSG technique/methodology. A parent or caregiver slept in the same room but in separate beds during both nights’ study. PSG studies were recorded by Rembrandt Med Care Diagnostics (Embla, Broomfield, CO, USA) for both groups of children. Non-autistic children were evaluated only for one night with standard four channel EEG for PSG. The rest of the PSG methodology was the same for both the ASD and non-autistic children. The start time for the recording of the PSG (Lights Off) was determined based on the child’s average bed time for both ASD and non-autistic children. All the PSG records of children with ASD were scored and reviewed by both XM and YMS (each record was scored twice), the PSG records of non-autistic children were reviewed by both XM and RVN in the same manner, using standard Rechtschaffen and Kales criteria.^15^ No significant disagreement was found between the scorers. In case of minor disagreement, a senior sleep medicine specialist was consulted and agreement was reached in these instances. Respiratory effort, airflow, end-tidal CO_2_ readings, oximetry readings, PLMS, EEG and EKG tracing were all scored by review of PSG recordings. Videos were reviewed simultaneously. Obstructive sleep apnea was defined as cessation of respiratory flow but retained respiratory effort for more than two respiratory cycles. Central apnea was defined as cessation of respiratory flow and effort for more than 20 seconds. Hypopnea was defined as a 50% or more reduction of respiratory flow accompanied by either an arousal or an oxygen desaturation of 4% from baseline.^16^,^17^ Tachycardia was defined as a heart rate greater than 100 beats per minutes or a 20% increase over the average heart rate during sleep. Parasomnia was determined by PSG findings, video and audio analysis, technician’s and on site parental reports. RBD was defined as a history or video evidence of acting out dreams (in the cases of children who can verbalize) combined with loss of REM atonia or increased phasic activities during REM.

There was no significant difference in general in sleep architecture on the two nights of recording in the children with ASD. Therefore, quantitative values for both nights of the PSG of children with ASD were averaged. As for questionnaire data, only frequently or weekly occurring historical parasomnias or snoring were included as positive values for reporting or analysis.

Statistical analysis was conducted in SAS (Version 8.0) and included two sample t-tests for continuous variables, Pearson Chi square and Fisher exact tests were utilized where proportions were being compared.

This study was approved by the Institutional Review Board of JFK Medical Center and Palisades Medical Center. Informed consent was obtained from parents/guardians of all subjects.

## Results

### Historical symptoms of sleep disorders

The sleep disorder symptoms abstracted from the questionnaires of both groups were recorded and summarized in Table 2. Twenty-one parents of the ASD children provided information regarding parasomnia, 16 answered “yes” to the questions regarding parasomnia (including questions on confusional arousal, sleep terror, sleep walking, bruxism and enuresis). The caregivers of five ASD children were uncertain of the nature of their child’s behavior upon awakening and therefore could not give definitive information. The remaining 2 parents of the ASD children left the questions pertaining parasomnia unanswered. Sixteen of the children with ASD had symptoms compatible with parasomnia: 10 with Disorders of Partial Arousal (sleep terror, confusional arousal, or sleepwalking), 7 with bruxism, and 7 with enuresis. For purposes of data analysis, one child was allowed to have none, one, or more than one parasomnias. Twenty two parents of the 23 non-autistic parents were able to complete the questions about parasomnia. Fifteen of the non-autistic children had a history compatible with parasomnias: Fourteen of them had bruxism and 2 had enuresis (one child had both). Of particular interest, when an adequate history was available, none of the parents of the non-autistic children reported that their child had a history compatible with a Disorder of Partial Arousal as compared to parental reports of that in 10 of 16 children with ASD. Also of importance, the age of the 7 ASD children with enuresis ranged from 7 to 15 years old, except for one 5 year old child. All the 7 ASD children did not wear diapers during the daytime. Two of these ASD children had a clinical history of epilepsy. Of the two non-autistic children who had a history of bedwetting, one was age 7, the other 4 years old.

More than half of the children with ASD and all the non-autistic children had symptoms of sleep disordered breathing. All the non-autistic children had a history of snoring because of the way the sample was selected (See Methods). Two of the non-autistic children had a history of both snoring and “stopping breathing during sleep”. Twenty one of the 23 children with ASD but only one of the non-autistic children had symptoms of sleep initiation and/or maintenance problems.

### Polysomnographic analysis of sleep disorders

The disparity of PSG finding of sleep parameters in ASD between the first and second night varies from subject to subject, among which the REM sleep percentage and periodic limb movements during sleep (PLMS) appear to be least disparate.

#### Parasomnia

Of the 14 children with ASD who had PSG evidence of parasomnia, 11 of them had a history suggestive of parasomnia by questionnaire: 10 children had a history of night crying or screaming, 4 children had a history of multiple episodes of crying or screaming within one single night at home (Table 4). The remaining three ASD children who had parasomnia in the laboratory had no complaint of parasomnia by questionnaire. On the other hand, 5 of the 23 ASD children had a history of parasomnia, but there was no behavior consistent with parasomnia during the PSG study.

The results of systemic analysis of polysomnographic records of non-autistic and ASD children are shown in Table 3. The most important finding is that children with ASD had significantly more parasomnias than those children in non-autistic comparison group (p = 0.002, Fisher’s exact test), and the reported parasomnia in children at large (1%–3%).^18^ Three non-autistic children’s videos were of insufficient clarity and were deemed to be missing. Fourteen ASD children had PSG evidence of parasomnia, while only 3 of the 20 non-autistic children had parasomnia in the laboratory. Of the 14 ASD children with parasomnia in the laboratory, 13 had a behavior compatible with Disorder of Partial Arousal (confusional arousal or sleep terror) and only one had bruxism recorded in PSG (Table 4). Furthermore, multiple episodes of partial arousal occurred in 11 of the 13 ASD children who had PSG evidence of Disorders of Partial Arousal. Of the 11 ASD children with multiple episodes of partial arousal, 6 ASD children had multiple partial arousals during both nights’ PSG study. The remaining one ASD child had bruxism in the laboratory. No child had evidence of RBD on either night of the PSG study as judged by behavioral, and tonic or phasic REM characteristics. Of the three non-autistic children who had parasomnias during PSG studies aged 3, 3 and 4 years old, two had bruxism and one had a Disorder of Partial Arousal consistent with Sleep Terror. All of these non-autistic children had a history compatible with a parasomnia (all three had a history suggestive of bruxism, one of the 3 children had an additional history suggestive of bedwetting) based by the questionnaire. However none of the three non-autistic children had a history compatible with a Disorders of Partial Arousal.

None of the ASD or non-autistic children was documented to have bedwetting during PSG studies.

Due to the high prevalence of parasomnia in ASD in this PSG study, we determined whether the parasomnia in ASD could be reliably diagnosed by questionnaire. Responses to parasomnia questions were associated with the presence or absence of parasomnia in PSG (Pearson Chi-Square test). The positive predictive value of parasomnia by questionnaire is 84.6%, however the negative predictive value is only 14%. The sensitivity is 11/17 (64.7%) while the specificity is 1/3 (33.3%).

The children with ASD who had partial arousal documented by PSG had concomitant autonomic arousal, manifested as tachycardia and increased respiratory rate and amplitude, as well as arm and leg movements as shown by video. Technician’s report and the videotape showed these children were confused during the arousals primarily from slow wave sleep. The parents or caregivers at bedside during PSG identified confusion of the child during these arousals. However, it was difficult to further determine the behaviors as to which of the two subtypes of the Disorders of Partial Arousal was present, i.e. sleep terror or confusional arousals. The other subtype of Disorders of Partial Arousal, sleep walking, was not seen in any of the ASD children. There was no EEG evidence (full seizure montage) of abnormal epileptiform activity prior, during or after the arousal.

We analyzed whether sleep disordered breathing in the small minority of the ASD children contributed to the increased prevalence of parasomnia in this cohort of ASD children. Parasomnia was not associated with higher AHI (AHI > 5events/hour, Fisher’s exact test, DF = 1, p = 1.0). We then analyzed whether age played a role in the frequency of parasomnia in children with ASD. The median age for children with ASD and parasomnia was 7 years, while the median age for ASD children without parasomnia was 6 years, suggesting that age was not a mere factor contributing to the increased frequency of parasomnia in this cohort of children with ASD (p = 0.596, DF = 14, Two-sample t test).

#### Sleep architecture

Sleep architecture was different in ASD children as compared with the non-autistic children. The arousal index was significantly higher in children with ASD than the non-autistic children (see Table 3, p = 0.004, t-test). The arousals were predominantly spontaneous in both groups. This finding is consistent with the higher prevalence of Disorders of Partial Arousal in the ASD group. The mild sleep disordered breathing in ASD did not contribute significantly to the arousals. The sleep latencies in ASD children were shortened. However this shorter sleep latency was probably caused by a later “Lights Out” in children with ASD as compared with non-autistic children. The median “Lights Out” time for non-autistic group in the lab was 9:38 PM, while the median “Lights Out” time for the ASD group in the lab was 10:58 PM. Therefore the shortened sleep latency in ASD could be a false positive. The median time for the termination of PSG “Lights On” in non-autistic group was 6:09 AM, while the median “lights on” time in autistic group was 6:12 AM. The total sleep time was therefore artificially shortened in some ASD children. However, one ASD child awakened at 1:30 AM and did not resume sleep during the first night’s study; his wake up time was 6:37 AM on the second night’s study. Five other ASD children had early awakening ranging form 4:23 AM to 5 AM that occurred randomly on the first or second night of the study. As a group, the percent of REM sleep was significantly reduced in ASD children (see Table 3, p = 0.002, Two Sample t-test). To determine whether the reduced total sleep time (TST) played a factor in REM percentage, we analyzed the association between TST and REM percentage. There was no significant association between TST and REM percentage (Pearson’s Chi Square test, DF = 1, p = 0.1). The mean REM latency was delayed in ASD children despite later sleep onset in the ASD group in comparison to the non-autistic group. However the difference was not statistically significant. The alterations of NREM sleep were variable among subjects of both groups and there appeared to be no consistent findings.

#### Sleep disordered breathing

There was an increase in sleep disordered breathing in children with ASD as evidenced by an increase in apnea hypopnea index (AHI). We did not compare the AHI in ASD children with this group of non-autistic children, as the non-autistic group was artificially selected to have a low AHI. However, the common consensus for a normal AHI in this age group of children is less than one apnea/hypopnea per hour. The mean AHI in children with ASD and the high prevalence of snoring during PSG (see Table 3) suggest that mild obstructive sleep apnea was common in this cohort of children with ASD. Four autistic children had a significant obstructive sleep apnea (AHI of 5.8, 8.5, 20.9 and 6.1 events per hour respectively).

#### Periodic Limb movement during sleep

PLMS were not a significant finding in either group of children. The average PLMS indices in both groups were less than 5 events per hour of sleep. There were no significant arousals associated with the PLMS in any of the children who exhibited PLMS.

#### Possible confounding factors

Fifteen of the autistic children and 7 non-autistic children used medication prior to PSG studies. Of those 15 ASD children, six children discontinued their medication completely: Three ASD children discontinued clonidine and zolpidem but remained on anticonvulsant medications (topiramate, valproate or oxicarbamazepine) during PSG studies. The rest 6 ASD children remained on the dopamine antagonist risperadone or aripiprazole, nasal steroid, or montelukast sodium. The seven non-autistic children used bronchodilator or nasal steroids on the day of PSG. We analyzed whether use of any medication contributed to the increased parasomnias. The result showed that medication use in general was not associated with the increased parasomnia in this cohort of ASD or non-autistic children (p = 0.65, Fisher’s exact test). We do not have a sufficient subject number to determine the impact of a specific medication on parasomnias.

All the 23 children with ASD had a history of one or more medical or behavioral comorbid disorders including gastrointestinal dysfunction (10 subjects), epilepsy (2 subjects), immune dysregulation (3 subjects), ADHD (6 subjects), anxiety disorder (4 subjects), and mood instability (7 subjects). No significant association was found between parasomnia and any one of the above comorbidities. Insufficient ASD subject numbers may have contributed to the insignificant statistical results.

## Discussion

This study is one of few studies that employed both questionnaire and polysomnographic data to investigate sleep disorders in ASD children and non-autistic children. The most significant finding is high prevalence of parasomnia in ASD children, especially the Disorders of Partial Arousal, both by questionnaire and PSG. Fourteen of the 23 children with ASD had PSG evidence of parasomnia (60.8%), 11 of these 14 ASD children had a history suggestive of parasomnia by questionnaire. Ten of the 18 ASD parents answered affirmatively to questions of Disorders of Partial Arousal (55.6%) of their child, and 13 of the 23 ASD children had PSG evidence of Disorders of Partial Arousal (56.5%). Multiple episodes of partial arousal occurred within the same night on both nights’ study in 11 of the 13 ASD children who had PSG evidence of Disorders of Partial Arousal. The sensitivity of the questionnaire in identification of parasomnia is reasonably high; however, the specificity is low. As a possible explanation for these results, we would comment that parasomnia may not occur nightly and there are sampling effects on the detection of parasomnia by PSG. In addition, sleep architecture was abnormal in ASD children, characterized by a significant increase of spontaneous arousals and a reduced REM percentage. Furthermore, there was an overall increase of mild sleep disordered breathing with exception of four children who had significant sleep disordered breathing.

In order to make comparable comparison with reported studies by others, we exercised two approaches as following. First, we calculated the prevalence of Disorders of Partial Arousal in sleep disordered patients selected from the general population (community children and ASD) based on the reported prevalence of subjects with sleep disorders and the prevalence of Disorders of Partial Arousal. The prevalence of Disorders of Partial Arousal of general community children with sleep problems ranges from 4.4%–17%.^19^,^20^,^21^ Paavonen et al. (2000) reported a prevalence of sleep disorders of 21.7% and night terror of 3.7% in 5813 Finnish normal 8–9 year old children. The adjusted night terror prevalence in the children with sleep disorders was 17%.^19^ Cai et al.^21^ reported a prevalence of sleep disorders 40.9% and a sleep terror of 1.8% in 3756 unselected urban community children at ages of 2–12 years. As adjusted, the sleep terror prevalence was 4.4% in the children with sleep disorders. The prevalence of Disorders of Partial Arousal in ASD with sleep complaints was higher. Liu et al.^22^ revealed a prevalence of 86% of sleep disorders and 20.4% of Disorders of Partial Arousal in survey of a group of unselected autistic children. As such, the prevalence of Disorders of Partial Arousal in the autistic children with sleep complaints was calculated to be 23.7%. Patzold et al.^23^ using questionnaire study in a general autistic children cohort, found an overall prevalence of sleep disorder was 63.2% and the prevalence of parasomnia (nightmare and enuresis, no data on Disorders of Partial Arousal) was 23%. The calculated prevalence of parasomnia in the sub-population of autistic children was 36.3%. Thus, our finding of high prevalence of parasomnia (especially Disorders of Partial Arousal) in ASD children with sleep complaints is consistent with other reported studies.

Our second approach is to extrapolate our findings of high prevalence of Disorders of Partial Arousal/parasomnia and apply to the general ASD population from which this smaller cohort originated from. We reported the prevalence of sleep disorders in ASD was 52%^24^ and current study showed a prevalence of Disorders of Partial Arousal of 55.6% by questionnaire in this sleep disordered ASD population. Therefore the prevalence of Disorders of Partial Arousal in our general ASD children is 28.9%, consistent with the reported prevalence in general ASD children.^22^,^23^ Similarly, our current study showed a prevalence of Disorders of Partial Arousal of 56.5% by PSG in this sleep disordered ASD population with a calculated prevalence of 29.4% for the general ASD population.

This current study is unique because it investigated the prevalence of sleep disorders using both questionnaire and PSG in ASD children with sleep complaints. In addition, this study reports a reasonable high sensitivity of the questionnaire in predicting PSG findings, especially of Disorders of Partial Arousal. There are only a limited number of studies utilizing both PSG and questionnaires^8^,^9^,^10^ focusing on sleep architecture and insomnia. None of these studies report prevalence of parasomnia. Miano et al.^8^ reported enuresis by questionnaire, but none by PSG. These investigators found a poor correlation of insomnia reported by questionnaire and the laboratory sleep staging.

The cause(s) of increased Disorders of Partial Arousal in ASD children is/are not clear. It is possible the increased sleep fragmentation in the ASD children predisposed them to the increased partial arousals. The stress and anxiety associated with sleep in the laboratory may contribute to the increased parasomnia in ASD children. It is reported that confusional arousals are strongly associated with the presence of a mental disorder with an odds ratios ranging from 2.4 to 13.5. Bipolar and anxiety disorders were the most frequently associated mental disorders.^25^ Mood disorder and anxiety disorders are frequently associated with ASD.^24^ It is possible that these comorbic psychiatric disorders (see Table 1) and mental retardation in the ASD children who participated in this study play a role in the increased prevalence of parasomnia. Confusional arousal is reported to be associated with brain lesions in area involving arousal such as the posterior hypothalamus, midbrain reticular formation, and periventricular gray matter.^26^ Abnormality of basal forebrain and brainstem has been implicated in autism.^27^,^28^,^29^ Dysfunction of neuronal network subserving arousal and slow wave sleep stability could be another possible etiology for the increased Disorders of Partial Arousal in ASD. Medications that alter the neurotransmission of arousal activity may also contribute to the increased occurrence of parasomnia. Unfortunately, the medications the ASD children were taking during this study were diverse and it is not possible to draw a conclusion of the impact of a specific medication on the increased parasomnia. However, medication use as a whole is not associated with parasomnia in this study. Another possibility is the contribution of sleep disordered breathing to parasomnia. However statistical analysis showed no association between sleep disordered breathing and parasomnia in this group of ASD children.

In contrast to the report of Thirumalai et al.^11^ this study failed to detect REM Sleep Behavior Disorder (RBD) in any of the subjects studied. Consistent with this study, Limoges et al.^7^ (2005) performed polysomnographic studies in 16 adults with high functioning autism and failed to reveal RBD. The apparent discrepancy with the report of Thirumalai et al. can perhaps be explained by different criteria applied in the diagnosis of RBD. We used stricter criteria that include both PSG and behavioral description of dream enactment in children who can verbalize. We did not detect loss of REM atonia in any of our subjects. This could also be due to different cohorts of subjects with ASD (apparent low developmental level, younger age in the study of Thirumalai et al.).^11^ Further extended sleep studies in ASD will hopefully shed light on this discrepancy.

This study demonstrates that parasomnias are prevalent in ASD children and clinical history of or questionnaire on parasomnia is sensitive in detecting parasomnia. Disorders of Partial Arousal may at least in part explain the reported sleep disruption, night crying or “awakening”, awakening with screaming, or “disoriented on waking” in ASD by questionnaire studies.^4^,^30^,^31^ Prevention and treatment of precipitating factors such as sleep deprivation, physical or psychological stress, illness may reduce the incidence of parasomnias in ASD children. In addition, sleep complaints in ASD children should be evaluated, at least by questionnaire, and treated accordingly.

The potential mechanism for our finding of mild obstructive sleep apnea in ASD could be associated with the reported reduced parasympathetic activity in ASD,^32^ leading to a reduced upper airway competency.

This study has its inherent limitations. This group of ASD children was selected based on their sleep disorder complaints that included symptoms compatible with parasomnia, the results of this study may not apply to the ASD population in general. The EEG montage, the one night versus two night studies, and different” Lights Out” times may have contributed in part to some of the abnormal sleep architectures and/or higher prevalence of parasomnia in ASD children. In the ASD group, the diagnosis, cognitive capacity, and the comorbidity were heterogeneous. Therefore the findings can not be applied to one specific subgroup of ASD. Increase ASD subject number and improvement in the design of future studies will hopefully address the limitations.

In summary, this study demonstrates that the prevalence of parasomnia especially Disorders of Partial Arousal is high in ASD children. Clinical history of parasomnia is reasonably reliable in diagnosing parasomnia when PSG is not an option. Future studies focusing on the effects of comorbic medical and psychiatric disorders on sleep disorders in ASD and the treatment of the comorbic disorders in improvement of the sleep disorders will hopefully bring relief to many ASD individuals and families suffering from the adverse effects of sleep disorders.

## Figures and Tables

**Table 1:** Characteristics of control and ASD subjects.^1^

	**Control**	**ASD**
Age (years)	3–12, Median 5	3–15, Median 6
Sex	8F, 15M,	4F, 19M
Primary Diagnosis^2^	Reactive airway disease and/or allergy: 7	Autistic disorder: 10
Comorbidity^2^	None	PDD-NOS: 11 Asperger’s disorder: 2 GI dysfunction: 10 Epilepsy: 2 Immune dysfunction: 3 ADHD: 6 Anxiety disorder: 4 Mood disorder: 7
Medication^3^	albuterol (5), nasal steroid (2)	topiramate (1), valproate (1), risperidone (2), aripiprazole (1), nasal steroid (2), montelukast sodium (3), oxcarbamazepine (1)

1 The total n-umber of subjects for each group is 23.

2 The numbers represent the number of subjects with the corresponding diagnosis.

3 The medication used during PSG studies. The number in the parentheses presents the number of subject taking the medication.

**Table 2:** Historical analysis of sleep disorders in ASD and controls.^1^

	**Control**	**ASD**
Snoring	23/23	13/23
Problems with Sleep Initiation and Maintenance	1/23	21/23
Parasomnia in General	15/23	16/23
*Bruxism*	14/23	7/19 *(4 unknown)*
*Disorders of Partial Arousal*	0/22 *(1 unknown)*	10/18 *(5 unknown)*
*Enuresis*	2/21 *(2 unknown)*	7/19 *(4 unknown)*

1 The numbers in the numerators are the subjects whose parents responded positively. The numbers in the denominators are the total numbers of subjects whose parents answered the corresponding questions. Numbers in the parentheses are the subjects whose parents were not able to answer to the questions.

**Table 3:** Polysomnographic analysis of sleep disorders in ASD and controls.^1^

	**Control**	**ASD**
Snoring	23/23 (100%)	17/22 (77%)
Arousal index^2^	6.0 ± 4.4	13.2 ± 8.8^*^
Sleep latency	22.4 ± 13.9 min	16.8 ± 22.0 min
Total sleep time	451 ± 59 min	395 ± 94 min
%REM	20.4 ± 4.6	14.2 ± 7.7^**^
REM latency	114 ± 47 min	146 ± 106 min
Parasomnias^3^	3/20	14/23^***^
PLMI	2.4 ± 5.9 (5/23)	2.45 ± 2.4 (19/23)

1 The total number was 23 each for controls and ASD children.

2 All the indices are events per hour.

3 The videos of three control subjects were not of good quality and deemed to be missing.

* t-test showed significantly difference (p = 0.004).

** Two sample t-test showed a significant difference (p = 0.002).

*** Fisher’s exact test showed a significant association of parasomnias with the ASD group (p = 0.002).

**Table 4:** Characteristics of parasomnia in asd children with sleep complaints.

**Number of children with parasomnia^1^**	**PSG**	**History**
Parasomnia (total)	14	16
Partial arousals	13	10
Multiple partial arousals	11	4
Multiple partial arousals on both nights	6	Not applicable
Bruxism	1	7
Enuresis	0	7

1 The total number of ASD children participated in the data analysis was 23. The numbers in the table represent the number of children who had the specific parasomnia.
